# Author Correction: Full reconstruction of simplicial complexes from binary contagion and Ising data

**DOI:** 10.1038/s41467-022-31470-6

**Published:** 2022-06-27

**Authors:** Huan Wang, Chuang Ma, Han-Shuang Chen, Ying-Cheng Lai, Hai-Feng Zhang

**Affiliations:** 1 The Key Laboratory of Intelligent Computing and Signal Processing of Ministry of Education, School of Mathematical Science, Anhui University, Hefei, 230601 China; 2 School of Internet, Anhui University, Hefei, 230601 China; 3 School of Physics and Material Science, Anhui University, Hefei, 230601 China; 4 School of Electrical, Computer and Energy Engineering, Arizona State University, Tempe, AZ 85287 USA

**Keywords:** Applied mathematics, Complex networks

Correction to: *Nature Communications* 10.1038/s41467-022-30706-9, published online 01 June 2022.

The original version of this article stated Chuang Ma as an equally contributing author in the Authors contributions, but omitted to mark it in the author list. This has now been corrected in both the PDF and HTML version of the article.

