# Supplementary material for: Full reconstruction of simplicial complexes from binary contagion and Ising data
Source: Nat Commun. 2022 Jun 1;13:3043. doi: 10.1038/s41467-022-30706-9 (PMC9160016; doi:10.1038/s41467-022-30706-9)
Supplement: Supplementary file 1 — Supplementary information [file 41467_2022_30706_MOESM1_ESM.pdf]

Supplementary Information for

Full reconstruction of simplicial complexes from binary contagion and Ising data

Huan Wang, Chuang Ma, Han-Shuang Chen, Ying-Cheng Lai, and Hai-Feng Zhang

Corresponding author: Hai-Feng Zhang (haifengzhang1978@gmail.com)

CONTENTS

I. Reconstruction accuracy under different epidemic conditions 2

II. Comparison of one-step method and two-step method 3

III. Reconstructing 2-simplicial complexes from simplicial Ising dynamics 3
A. Binary data from simplicial Ising dynamics 3
B. Statistical inference framework for simplicial Ising dynamics 4
C. Representative reconstruction results for simplicial Ising dynamics 7

IV. Reconstruction performance in terms of the number of events 8

V. O-information measure 8

VI. Effect of time-series length on reconstruction performance 9

VII. Supplementary figures 10

VIII. Supplementary references 22

I. RECONSTRUCTION ACCURACY UNDER DIFFERENT EPIDEMIC CONDITIONS

In general, the reconstruction accuracy depends on the epidemic conditions. In the main text, the parameter values for generating the data are selected near the epidemic threshold, i.e., near the triangle-driven and edge-driven transitions. To assess the effects of choosing parameter values in other regimes on the reconstruction results, we focus on two quantities: the rescaled edge infectivity α and the rescaled triangular infectivity ω, and study their impact on the reconstruction accuracy of two-body and three-body connections. In particular, the social contagion probabilities are $\beta_1 = \alpha/k_1$ and $\beta_2 = \omega/k_2$, where k_1 and k_2 are the average degrees of two-body and three-body connections in a 2-simplicial complex, respectively.

Different values of β_1 and β_2 can have significantly different impacts on the reconstruction results. For large values of β_1 or β_2, many nodes are infected, making it difficult to judge which infected nodes have spread the infection to the node under reconstruction. On the contrary, for small values of β_1 and β_2, it is difficult for a susceptible node to be infected by other nodes, leading to a lack of the useful data for the reconstruction task. It is then necessary to select the values of β_1 and β_2 properly to achieve acceptable reconstruction accuracy. We have systematically investigated the different parameter settings (e.g., in terms of the rescaled infectivities α and ω) and their effects on the reconstruction accuracy.

To be concrete, we study a scale-free simplicial complex (SFSC) with $N = 200$, $k_1 = 14$ and $k_2 = 4$. Figure S1 shows the average fraction ρ^* of infected nodes in the stationary state versus α for different values of ω. It can be seen that, as ω increases, the nature of the phase transition in the underlying social contagion dynamics changes from continuous to discontinuous. Further, different initial values of the density ρ_0 of the infected nodes can affect the steady-state infection density ρ^* associated with the healthy and endemic states in the bistable region.

We then choose three different values of α (0.5, 0.8 and 1.5), which are below, near and above the edge epidemic threshold α_c, respectively, and investigate the effect of different values of ω on the reconstruction accuracy. Concretely, we choose $\omega = 0.8$, 2.4 and 4.0, which are below, near and above the triangular epidemic threshold ω_c, respectively. Figure S2(a) reveals that, for $\alpha = 0.5$, the reconstruction accuracies of two-body and three-body connections are not high, because it is difficult for social contagion in simplicial complexes to propagate when the rescaled edge infectivity α is small. Increasing the value of ω can lead to an improvement in the reconstruction accuracy. For example, as shown in Fig. S2(b), for $\alpha = 0.8$ and reasonably long time series, the reconstruction accuracies of two-body and three-body connections for $\omega = 2.4$ are the highest, as a small value of ω means an insignificant synergistic reinforcement effect from the three-body connections but a large value of ω will weaken the interactions from the two-body connections, especially when the value of α is near the edge threshold: both effects lead to difficulties in reconstructing two-body or three-body connections. Figure S2(c) shows, for $\alpha = 1.5$, the reconstruction accuracies of two-body and three-body connections for $\omega = 2.4$ are generally higher than those in the other two cases, because a small value of ω (e.g., $\omega = 0.8$) is not able to generate a strong synergistic reinforcement effect from the three-body connections while a large value of ω (e.g., $\omega = 4$) will cause most nodes to be infected. Taken together, the highest possible reconstruction accuracies are achieved when the values of α and ω are near their respective epidemic thresholds.

II. COMPARISON OF ONE-STEP METHOD AND TWO-STEP METHOD

In order to compare the accuracy and time complexity of one-step and two-step methods, we have used scale-free simplicial complex (SFSC) with different sizes and average degrees. The results are shown in Figs. S3 and S4. In particular, Fig. S3 shows that the accuracy of the two-step method is higher than that of the one-step method for both two-body and three-body reconstruction. The superiority of two-step method is more evident when the network size is larger. Figure S4 demonstrates the running time of the two methods, which are implemented in MATLAB2016a and run on a Linux machine with 2.60-GHz Intel processor, 28 CPU cores, and 192-GB RAM. It can be seen that the required computational time of the two-step method is more than one order of magnitude lower than that required of the one-step method.

III. RECONSTRUCTING 2-SIMPLICIAL COMPLEXES FROM SIMPLICIAL ISING DYNAMICS

We introduce a simplicial Ising model and demonstrate that our statistical inference framework for reconstructing 2-simplicial complexes based on the binary Ising data. The Ising model is fundamental in statistical physics and complex systems, which can be used to describe a variety of phenomena such as phase transitions, coarsening dynamics, and opinion formation.

A. Binary data from simplicial Ising dynamics

Let S_i^t be the state of node i at time t. Each node has two possible states: spin-down ($S_i^t = -1$) or spin-up ($S_i^t = +1$). For a 2-simplicial complex hosting simplicial Ising dynamics, the Hamiltonian is given by

$$H(t) = -J_1 \sum_{(i,j)} S_i^t S_j^t - J_2 \sum_{(i,j,k)} S_i^t S_j^t S_k^t, \tag{S3.1}$$

where J_1 and J_2 are the strengths of two-body and three-body interactions, and (i, j) and (i, j, k) denote the two-body and the three-body connections in the 2-simplicial complex, respectively. The first term in the Hamiltonian characterizes the interaction between the edges (i.e., two-body connections) and the second term contains three-body interactions from the 2-simplex. At each time step, the spin-flipping probability of each node i is given by

$$\mathrm{f}_i(t+1) = \frac{1}{1 + e^{\delta \Delta E_i^t}}, \tag{S3.2}$$

in which δ is the inverse temperature. The quantity

$$\Delta E_i^t = 2J_1 \sum_{(i,j)\in\partial_i} S_i^t S_j^t + 2J_2 \sum_{(i,j,k)\in\nabla_i} S_i^t S_j^t S_k^t$$

represents the change in the energy caused by a flipping of node i at time t, where ∂_i and ∇_i are the 1-simplex set and the 2-simplex set containing node i, respectively. At the initial time, the state of each node i is randomly assigned as $+1$ or -1 with equal probability. Binary time series are generated according to Eq. (S3.2) and the data are stored in the data matrix S.

B. Statistical inference framework for simplicial Ising dynamics

Similar to the case of the social contagion dynamics in the main text, we use the two-step reconstruction strategy to reconstruct 2-simplicial complex for simplicial Ising dynamics. At the first step, the "approximate" neighbors of each node are predicted and their corresponding columns in the data matrix S are extracted. At the second step, based on the compressed data matrix, the final neighbors and the 2-simplex of node i are determined. The main processes of step 1 and step 2 are as follows.

Let C and $\bar{C}$ represent the two distinct states of nodes, through which the transition from "+1" to "-1" and that from "-1" to "+1" can be described in a common setting. For the first step, according to the Bayesian formula, the conditional probability of $S_i^{t+1} = \bar{C}$ and $j \to i$, i.e., the event that node j has a direct impact on the state of node i given $S_i^t = C$ and $S_j^t = \bar{C}$, can be written as

$$\begin{aligned} &\mathrm{P}\left(S_i^{t+1} = \bar{C}, j \to i \left| S_i^t = C, S_j^t = \bar{C}\right.\right) \\ &= \mathrm{P}\left(j \to i \left| S_i^t = C, S_j^t = \bar{C}, S_i^{t+1} = \bar{C}\right.\right) \mathrm{P}\left(S_i^{t+1} = \bar{C} \left| S_i^t = C, S_j^t = \bar{C}\right.\right) = \mathrm{P}_{j\to i}^0 \mathrm{P}_j^i, \end{aligned} \tag{S3.3}$$

where

$$\mathrm{P}_{j\to i}^0 = \mathrm{P}\left(j \to i \left| S_i^t = C, S_j^t = \bar{C}, S_i^{t+1} = \bar{C}\right.\right)$$

denotes the probability that node i changes from C state to $\bar{C}$ state due to the excitation of node j, under the conditions $S_i^t = C$, $S_j^t = \bar{C}$ and $S_i^{t+1} = \bar{C}$. Theoretically, $\mathrm{P}_{j\to i}^0 > 0$ indicates that node j is an "approximate" neighbor of node i; otherwise, there is no edge connecting nodes i and j. The quantity

$$\mathrm{P}_j^i = \mathrm{P}\left(S_i^{t+1} = \bar{C} \left| S_i^t = C, S_j^t = \bar{C}\right.\right)$$

is the probability of $S_i^{t+1} = \bar{C}$ under the conditions $S_i^t = C$ and $S_j^t = \bar{C}$, which can be estimated from the data matrix S.

From Eq. (S3.3), the expected number of node i in $\bar{C}$ state at $t_m + 1$ can be expressed as

$$\tilde{\mathrm{E}}_i^{t_m+1} = \sum_{j(j\neq i)} \mathrm{P}\left(S_i^{t_m+1} = \bar{C}, j \to i \left| S_i^{t_m} = C, S_j^{t_m} = \bar{C}\right.\right) \Psi_j^{t_m} + \varepsilon_i = \sum_{j(j\neq i)} \mathrm{P}_{j\to i}^0 \mathrm{P}_j^i \Psi_j^{t_m} + \varepsilon_i, \tag{S3.4}$$

where $\Psi_j^{t_m}$ represents the expected times of node j in $\bar{C}$ state at time t_m, and their values are zero or one. The quantity ε_i represents noise due to the errors from the collected data. Assuming Ψ_i of node i obeys Poisson distribution, we get the likelihood function as

$$\mathrm{P}\left(\left\{\Psi_i^{t_m+1}\right\}_{m=1,\cdots,M} \left| \tilde{\Theta}, \left\{\Psi_j^{t_m}\right\}_{m=1,\cdots,M; j=1,\cdots,N}\right.\right) = \prod_{m\left(\Psi_i^{t_m}=C\right)} \frac{e^{-\tilde{\mathrm{E}}_i^{t_m+1}} \left(\tilde{\mathrm{E}}_i^{t_m+1}\right)^{\Psi_i^{t_m+1}}}{\Psi_i^{t_m+1}!}, \tag{S3.5}$$

where $\tilde{\Theta}$ denotes the set of variables $\mathrm{P}_{j\to i}^0$ and ε_i. We have $\Psi_i^{t_m+1}! \equiv 1$ since $\Psi_i^{t_m+1}$ is either zero

or one. Taking the logarithm of Eq. (S3.5), we have

$$L\left(\tilde{\Theta}\right) = \sum_{m\left(\Psi_i^{t_m}=C\right)} \left(\Psi_i^{t_m+1}\log\tilde{\mathrm{E}}_i^{t_m+1} - \tilde{\mathrm{E}}_i^{t_m+1}\right) = \sum_{m\left(\Psi_i^{t_m}=C\right)} \left[\begin{array}{l} \Psi_i^{t_m+1}\log\left(\sum\limits_{j(j\neq i)} \mathrm{P}_{j\to i}^0 \mathrm{P}_j^i \Psi_j^{t_m} + \varepsilon_i\right) \\ -\left(\sum\limits_{j(j\neq i)} \mathrm{P}_{j\to i}^0 \mathrm{P}_j^i \Psi_j^{t_m} + \varepsilon_i\right) \end{array}\right]. \tag{S3.6}$$

Using the expectation maximization (EM) method [1] to maximize the likelihood function for determining the parameter $\tilde{\Theta}$ in Eq. (S3.5), we have

$$\mathrm{P}_{j\to i}^0 = \frac{\sum\limits_{m\left(\Psi_i^{t_m}=C\right)} \left(\Psi_i^{t_m+1}\rho_j^{t_m}\right)}{\sum\limits_{m\left(\Psi_i^{t_m}=C\right)} \left(\mathrm{P}_j^i \Psi_j^{t_m}\right)}, \tag{S3.7}$$

$$\varepsilon_i = \frac{\sum\limits_{m\left(\Psi_i^{t_m}=C\right)} \left(\Psi_i^{t_m+1}\rho_{\varepsilon_i}^{t_m}\right)}{\sum\limits_{m\left(\Psi_i^{t_m}=C\right)} (1)}, \tag{S3.8}$$

where

$$\rho_j^{t_m} = \frac{\mathrm{P}_{j\to i}^0 \mathrm{P}_j^i \Psi_j^{t_m}}{\sum\limits_{j'\left(j'\neq i\right)} \mathrm{P}_{j'\to i}^0 \mathrm{P}_{j'}^i \Psi_{j'}^{t_m} + \varepsilon_i}, \tag{S3.9}$$

$$\rho_{\varepsilon_i}^{t_m} = \frac{\varepsilon_i}{\sum\limits_{j'\left(j'\neq i\right)} \mathrm{P}_{j'\to i}^0 \mathrm{P}_{j'}^i \Psi_{j'}^{t_m} + \varepsilon_i}. \tag{S3.10}$$

Equations (S3.7)-(S3.10) are the key formulas for finding the unknown quantities $\mathrm{P}_{j\to i}^0$ and ε_i. By initializing $\mathrm{P}_{j\to i}^0$ and ε_i and repeating the above four equations until convergence is achieved and using the same truncation method as in social contagion dynamics, we can determine the "approximate" neighbors of node i.

At the second step, similar to Eq. (S3.3) in the first step, the conditional probability of $S_i^{t+1} = \bar{C}$ and $j \to i$ given $S_i^t = C$ and $S_j^t = \bar{C}$ can be written as

$$\begin{array}{l} \mathrm{P}\left(S_i^{t+1} = \bar{C}, j \to i \left| S_i^t = C, S_j^t = \bar{C}\right.\right) \\ = \mathrm{P}\left(j \to i \left| S_i^t = C, S_j^t = \bar{C}, S_i^{t+1} = \bar{C}\right.\right) \mathrm{P}\left(S_i^{t+1} = \bar{C} \left| S_i^t = C, S_j^t = \bar{C}\right.\right) = \mathrm{P}_{j\to i}\mathrm{P}_j^i, \end{array} \tag{S3.11}$$

where

$$\mathrm{P}_{j\to i} = \mathrm{P}\left(j \to i \left| S_i^t = C, S_j^t = \bar{C}, S_i^{t+1} = \bar{C}\right.\right)$$

denotes the probability that node i changes from C state to $\bar{C}$ state due to the excitation of node j, under the conditions $S_i^t = C$, $S_j^t = \bar{C}$ and $S_i^{t+1} = \bar{C}$. Theoretically, $\mathrm{P}_{j\to i} > 0$ indicates that node j is a neighbor of node i; otherwise, there is no edge connecting nodes i and j. The quantity

$$\mathrm{P}_j^i = \mathrm{P}\left(S_i^{t+1} = \bar{C} \left| S_i^t = C, S_j^t = \bar{C}\right.\right)$$

is the probability of $S_i^{t+1} = \bar{C}$ under the conditions $S_i^t = C$ and $S_j^t = \bar{C}$, which can be estimated from the compressed data matrix.

Similarly, the conditional probability of $S_i^{t+1} = \bar{C}$ and $jk \to i$ (i.e., the event that the synergistic reinforcement effect coming from nodes j and k has a direct impact on the state of node i) given the conditions $S_j^t S_k^t = \bar{C}$ and $S_i^t = C$, can be written as

$$\begin{aligned} &\mathrm{P}\left(S_i^{t+1} = \bar{C}, jk \to i \,\middle|\, S_i^t = C, S_j^t S_k^t = \bar{C}\right) \\ &= \mathrm{P}\left(jk \to i \,\middle|\, S_i^t = C, S_j^t S_k^t = \bar{C}\ , S_i^{t+1} = \bar{C}\right) \mathrm{P}\left(S_i^{t+1} = \bar{C} \,\middle|\, S_i^t = C, S_j^t S_k^t = \bar{C}\right) \\ &= \mathrm{P}_{jk\to i}\mathrm{P}_{jk}^i, \end{aligned} \quad \text{(S3.12)}$$

where

$$\mathrm{P}_{jk\to i} = \mathrm{P}\left(jk \to i \,\middle|\, S_i^t = C, S_j^t S_k^t = \bar{C}, S_i^{t+1} = \bar{C}\right)$$

is the probability that node i changes from C state to $\bar{C}$ state because of the synergistic excitation from nodes j and k, under the conditions $S_i^t = C$, $S_j^t S_k^t = \bar{C}$ and $S_i^{t+1} = \bar{C}$. The quantity $\mathrm{P}_{jk\to i}$ being positive indicates that nodes i, j and k form a 2-simplex, otherwise, they do not form a 2-simplex. The probability

$$\mathrm{P}_{jk}^i = \left(S_i^{t+1} = \bar{C} \,\middle|\, S_i^t = C, S_j^t S_k^t = \bar{C}\right)$$

can be estimated from the compressed data matrix in a similar way.

According to Eqs. (S3.11) and (S3.12), the expected number of node i in $\bar{C}$ state at $t_m + 1$ is given by

$$\begin{aligned} \mathrm{E}_i^{t_m+1} &= \sum_{j(j\neq i)} \mathrm{P}\left(S_i^{t_m+1} = \bar{C}, j \to i \,\middle|\, S_i^{t_m} = C, S_j^{t_m} = \bar{C}\right)\Psi_j^{t_m} \\ &+ \sum_{j,k(j\neq k\neq i)} \mathrm{P}\left(S_i^{t_m+1} = \bar{C}, jk \to i \,\middle|\, S_i^{t_m} = C, S_j^{t_m} S_k^{t_m} = \bar{C}\right)\Psi_{jk}^{t_m} + \varepsilon_i \\ &= \sum_{j(j\neq i)} \mathrm{P}_{j\to i}\mathrm{P}_j^i\Psi_j^{t_m} + \sum_{j,k(j\neq k\neq i)} \mathrm{P}_{jk\to i}\mathrm{P}_{jk}^i\Psi_{jk}^{t_m} + \varepsilon_i, \end{aligned} \quad \text{(S3.13)}$$

where $\Psi_j^{t_m}$ represents the expected times of node j in $\bar{C}$ state at time t_m. Similarly, $\Psi_{jk}^{t_m}$ is the expected times of both nodes j and k being in $\bar{C}$ state at time t_m, and their values are zero or one. Assuming that the number Ψ_i in each time period obeys Poisson distribution, we obtain the likelihood function as

$$\mathrm{P}\left(\left\{\Psi_i^{t_m+1}\right\}_{m=1,\cdots,M} \,\middle|\, \Theta, \left\{\Psi_j^{t_m}\right\}_{m=1,\cdots,M;j=1,\cdots,N}\right) = \prod_{m\left(\Psi_i^{t_m}=C\right)} \frac{e^{-\mathrm{E}_i^{t_m+1}}\left(\mathrm{E}_i^{t_m+1}\right)^{\Psi_i^{t_m+1}}}{\Psi_i^{t_m+1}!}, \quad \text{(S3.14)}$$

where Θ denotes the set of variables $\mathrm{P}_{j\to i}$, $\mathrm{P}_{jk\to i}$ and ε_i.

Using the EM method to maximize the likelihood function for determining the parameter Θ in

Eq. (S3.14), we obtain

$$\mathrm{P}_{j\to i}=\frac{\sum\limits_{m\left(\Psi_i^{t_m}=C\right)}\left(\Psi_i^{t_m+1}\rho_j^{t_m}\right)}{\sum\limits_{m\left(\Psi_i^{t_m}=C\right)}\left(\mathrm{P}_j^i\Psi_j^{t_m}\right)},\tag{S3.15}$$

$$\mathrm{P}_{jk\to i}=\frac{\sum\limits_{m\left(\Psi_i^{t_m}=C\right)}\left(\Psi_i^{t_m+1}\rho_{jk}^{t_m}\right)}{\sum\limits_{m\left(\Psi_i^{t_m}=C\right)}\left(\mathrm{P}_{jk}^i\Psi_{jk}^{t_m}\right)},\tag{S3.16}$$

$$\varepsilon_i=\frac{\sum\limits_{m\left(\Psi_i^{t_m}=C\right)}\left(\Psi_i^{t_m+1}\rho_{\varepsilon_i}^{t_m}\right)}{\sum\limits_{m\left(\Psi_i^{t_m}=C\right)}(1)},\tag{S3.17}$$

where

$$\rho_j^{t_m}=\frac{\mathrm{P}_{j\to i}\mathrm{P}_j^i\Psi_j^{t_m}}{\left(\begin{array}{l}\sum\limits_{j'\left(j'\neq i\right)}\mathrm{P}_{j'\to i}\mathrm{P}_{j'}^i\Psi_{j'}^{t_m}+\\ \sum\limits_{j',k'\left(j'\neq k'\neq i\right)}\mathrm{P}_{j'k'\to i}\mathrm{P}_{j'k'}^i\Psi_{j'k'}^{t_m}+\varepsilon_i\end{array}\right)},\tag{S3.18}$$

$$\rho_{jk}^{t_m}=\frac{\mathrm{P}_{jk\to i}\mathrm{P}_{jk}^i\Psi_{jk}^{t_m}}{\left(\begin{array}{l}\sum\limits_{j'\left(j'\neq i\right)}\mathrm{P}_{j'\to i}\mathrm{P}_{j'}^i\Psi_{j'}^{t_m}+\\ \sum\limits_{j',k'\left(j'\neq k'\neq i\right)}\mathrm{P}_{j'k'\to i}\mathrm{P}_{j'k'}^i\Psi_{j'k'}^{t_m}+\varepsilon_i\end{array}\right)},\tag{S3.19}$$

$$\rho_{\varepsilon_i}^{t_m}=\frac{\varepsilon_i}{\left(\begin{array}{l}\sum\limits_{j'\left(j'\neq i\right)}\mathrm{P}_{j'\to i}\mathrm{P}_{j'}^i\Psi_{j'}^{t_m}+\\ \sum\limits_{j',k'\left(j'\neq k'\neq i\right)}\mathrm{P}_{j'k'\to i}\mathrm{P}_{j'k'}^i\Psi_{j'k'}^{t_m}+\varepsilon_i\end{array}\right)}.\tag{S3.20}$$

Taken together, the six equations Eqs. (S3.15)-(S3.20) can be used to solve $\mathrm{P}_{j\to i}$, $\mathrm{P}_{jk\to i}$, and ε_i by initializing $\mathrm{P}_{j\to i}$, $\mathrm{P}_{jk\to i}$, ε_i and repeating the above six equations until convergence is achieved.

We remark that, according to the flipping probability given by Eq. (S3.2), each node always has a large or small flipping probability regardless of the neighbors state at each time step, so noise is always present in the time-series data. As a result, the prediction of two-body connections at the second step is truncated according to Eq. (26) in the main text, instead of simply cutting from zero as in the case of social contagion dynamics. For predicting the three-body connections, the same truncation method as in the social contagion dynamics can be used.

C. Representative reconstruction results for simplicial Ising dynamics

Figures S5 and S6 present results on random simplicial complex (ERSC), scale-free simplicial complex (SFSC), and small-world simplicial complex (SWSC) for $N = 100$ and $N = 200$,

respectively. Reconstruction results from four real-world 2-simplicial complexes are shown in Fig. S7.

Similar to the case of reconstructing 2-simplicial complexes from the social contagion model, Figs. S5-S7 reveal that increasing the time-series length T can improve the reconstruction accuracy of two-body and three-body connections. In addition, a small value of k_1 tends to increase the reconstruction accuracies of both types of simplexes. However, different values of k_2 have little effect on the reconstruction accuracies. For the same amount of data, larger networks generally lead to lower accuracies. Finally, the reconstruction accuracy of two-body connections is generally higher than that of the three-body connections.

IV. RECONSTRUCTION PERFORMANCE IN TERMS OF THE NUMBER OF EVENTS

We present F1 score as a function of the number of events for scale-free simplicial complex (SFSC) (Fig. S8) and four real-world 2-simplicial complexes (Fig. S9), where the number of events is calculated on each node. For example, 100 events means that each node has 100 switches between susceptible and infected states. It can be seen that the reconstruction accuracy increases with the number of events. In addition, the reconstruction accuracies of two-body and three-body connections decrease with k_1 (i.e., average degree of 1-simplex), but the value of k_2 (i.e., average degree of 2-simplex) affects only the accuracy of three-body connections and has little effect on the accuracy of reconstructing two-body connections. While a larger simplicial complex requires more events to reach certain F1 score, with sufficient data a high reconstruction accuracy can still be achieved. The conclusion is that the results based on the number of events are essentially the same as those in terms of the simulation time T.

V. O-INFORMATION MEASURE

In the recent work [2], an important information metric named O-information (Ω) was introduced, which can be used to characterize synergy- and redundancy-dominated systems and quantify higher-order interdependencies. It can be seen from Lemma 1 in the paper that O-information can only capture the interactions that go beyond the pairwise relationships, so it is not suitable for describing the interaction of two variables, i.e., it cannot be used to reconstruct the pairwise relationships.

More Specifically, for a system of three discrete variables, the O-information is defined as

$$\Omega\left(X^{n}\right)=H\left(X^{n}\right)+\sum_{j=1}^{n}\left[H\left(X_{j}\right)-H\left(X_{-j}^{n}\right)\right],$$

where

$$\begin{aligned}H\left(X^{n}\right)&=-\sum_{X^{n}}P_{X^{n}}\left(X^{n}\right)\log P_{X^{n}}\left(X^{n}\right),\\X^{n}&=\left(X_{1},\cdots,X_{n}\right),\\X_{-j}^{n}&=\left(X_{1},\cdots,X_{j-1},X_{j+1},\cdots,X_{n}\right),\end{aligned}$$

for $n=3$. According to this metric, the system is redundancy dominated if $\Omega(X^{3})>0$; otherwise ($\Omega(X^{3})<0$), it is synergy dominated. To compare our method with this O-information based

method, we have calculated the O-information values between any three variables. If it is negative, there is an interaction among the three variables (three-body connection), otherwise the connection does not exist. The results are shown in Fig. S10, where the O-information values of any three points are displayed with the blue and red dots denoting the existent and nonexistent three-body connections, respectively. It can be seen that the O-information values associated with most of the existent three-body interactions are indeed negative, but the O-information values of many nonexistent three-body interactions are also negative, making it impossible to distinguish the two cases and to ascertain the existent three-body interactions.

Overall, the O-information method proposed in [PRE 100, 032305 (2019)] relies on strong correlations among the time series of the dynamical variables for predicting the synergy structure. In our case, the dynamical time-series data are obtained by alternating updated iterations, that is, the state transition of each node is determined by the states of neighbors at the previous time, leading to only weak correlations among different nodal pairs at any given time. As a result, the O-information method is not suitable for predicting higher-order structures from dynamical time-series data.

VI. EFFECT OF TIME-SERIES LENGTH ON RECONSTRUCTION PERFORMANCE

The reconstruction performance on synthetic and real-world 2-simplicial complexes versus the length T of the time series has been studied for different noise level (the flip ratio) f: $f = 0$ (without noise), 0.1, 0.2, and 0.3, with results shown in Figs. S11 and S12, respectively. These results indicate that, for short time series, the reconstruction performance is sensitive to noise. For example, when the value of f changes from 0 to 0.1, there is a sizable reduction in the F1 score. Regardless of the noise level, increasing the length of time series can always improve the reconstruction performance for both two-body and three-body interactions.

VII. SUPPLEMENTARY FIGURES

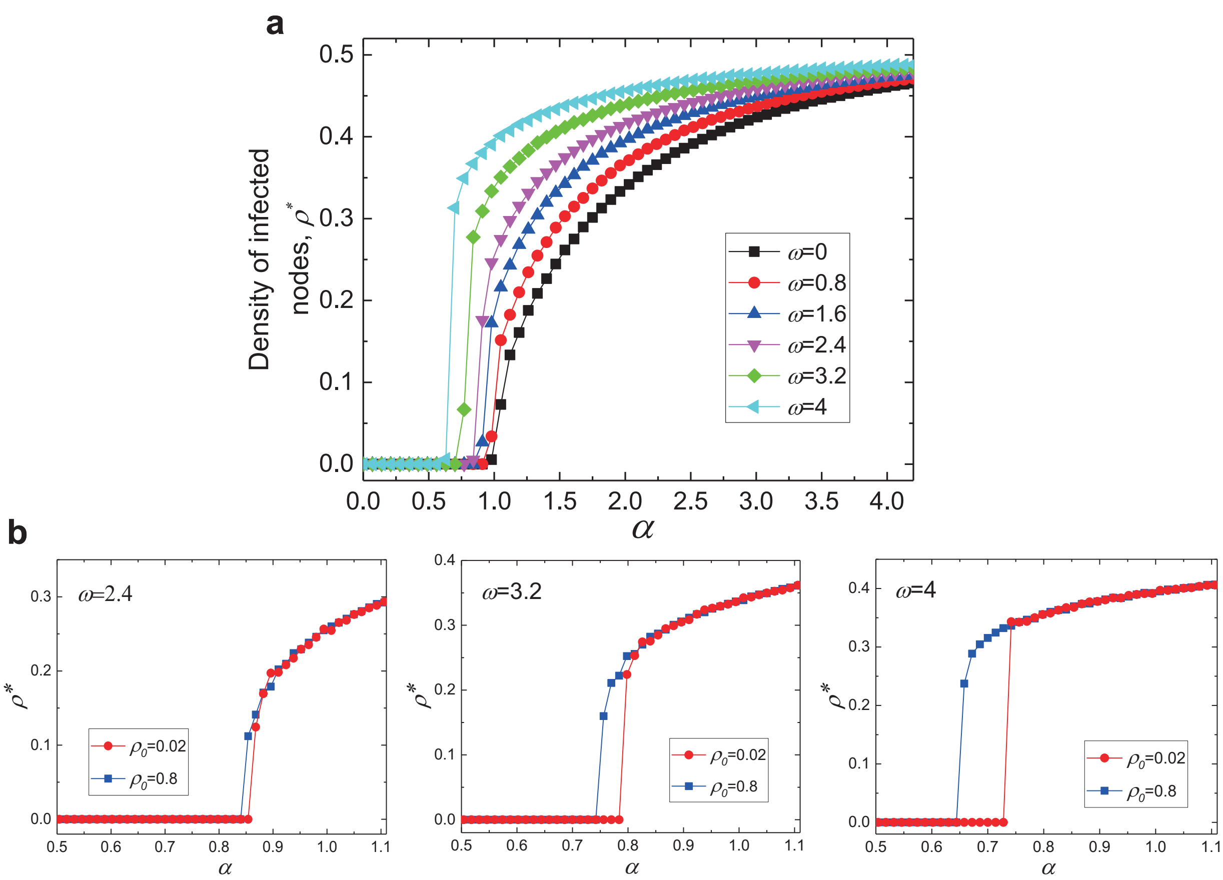

FIG. S1. Average fraction ρ^* of infected nodes in the stationary state as a function of the rescaled edge infectivity α for SFSC. (**a**) The resulting ρ^*-vs-α curves for different values of the rescaled triangular infectivity ω for $\rho_0 = 0.2$ and $\mu = 1$. As ω increases, the nature of the phase transition in the underlying social contagion dynamics changes from continuous to discontinuous. (**b**) The effect of the initial density of the infected nodes on the ρ^*-vs-α curve for three values ω (three subpanels). In each subpanel, the ρ^*-vs-α curves for two values of ρ_0 are shown: $\rho_0 = 0.02$ (red circles) and $\rho_0 = 0.8$ (blue squares). Different initial values of the density ρ_0 of the infected nodes can affect the steady-state infection density ρ^* associated with the healthy and endemic states in the bistable region. For all the curves in (**a**) and (**b**), each data point is the result of averaging over 50 statistical realizations.

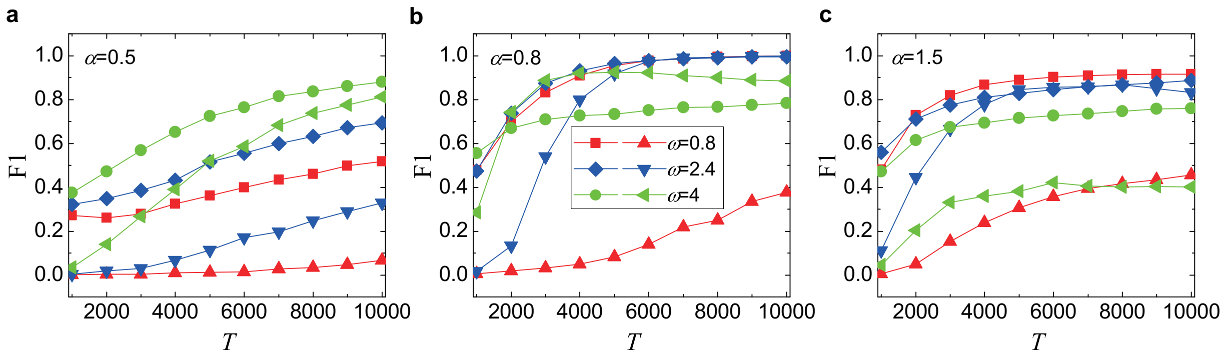

FIG. S2. Reconstruction performance for different values of α and ω. Shown is F1 score as a function of the time series length T for SFSC for (**a**) $\alpha = 0.5$, (**b**) $\alpha = 0.8$, and (**c**) $\alpha = 1.5$. In each panel, squares, diamonds and circles denote the accuracy of reconstructing two-body connections, while triangles with different orientations denote the accuracy of reconstructing three-body connections. The results from different values of ω are distinguished by colors. Other parameter values are $\rho_0 = 0.2$ and $\mu = 1$, and five realizations are used to generate the results.

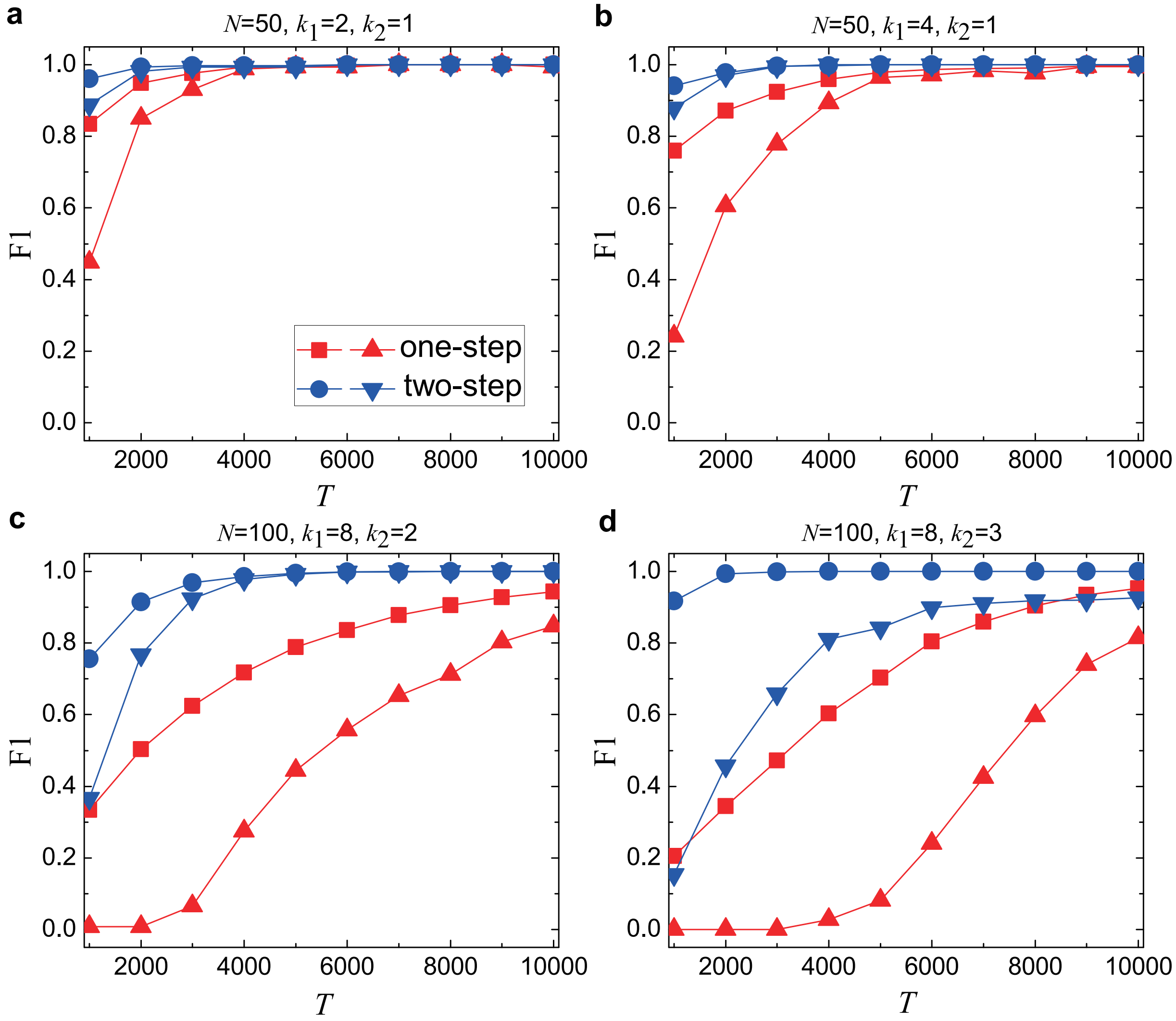

FIG. S3. Comparison of the reconstruction performance between one-step and two-step methods in SFSC with different sizes and degrees. Shown is F1 score as a function of the time-series length T. Squares and circles denote the performance of reconstructing two-body connections while triangles with different orientations are for reconstructing three-body connections. The results from the two methods are distinguished by colors. The parameter values in each simplicial complex are (**a**) N = 50, k_1=2, k_2=1, α=0.2, ω=1, (**b**) N=50, k_1=4, k_2=1, α=0.4, ω=1, (**c**) N= 100, k_1=8, k_2=2, α=0.6, ω=1.55, and (**d**) N= 100, k_1=8, k_2=3, α=0.8, ω=1.6. Other parameter values are $\rho_0 = 0.2$ and $\mu = 1$. The results are averaged over five realizations.

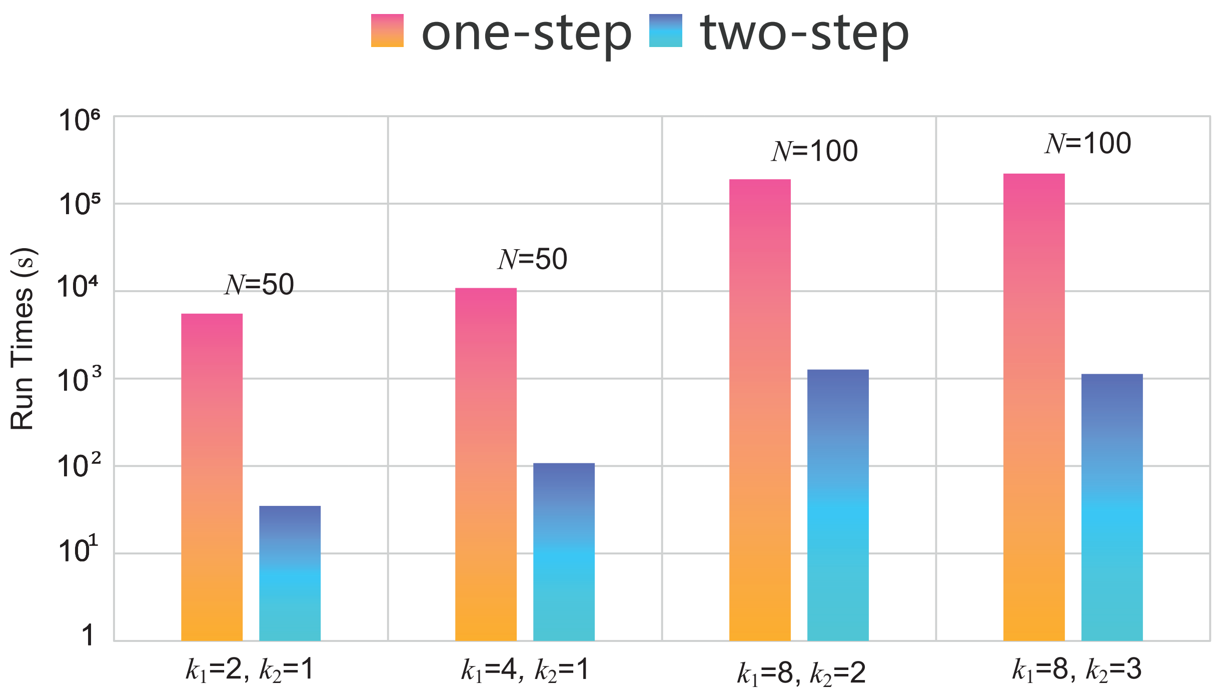

FIG. S4. Comparison of the required computational time of the one-step method and two-step method on SFSC with different sizes and degrees. The parameter values are the same as those in Fig. S3. The running time is calculated based on one realization.

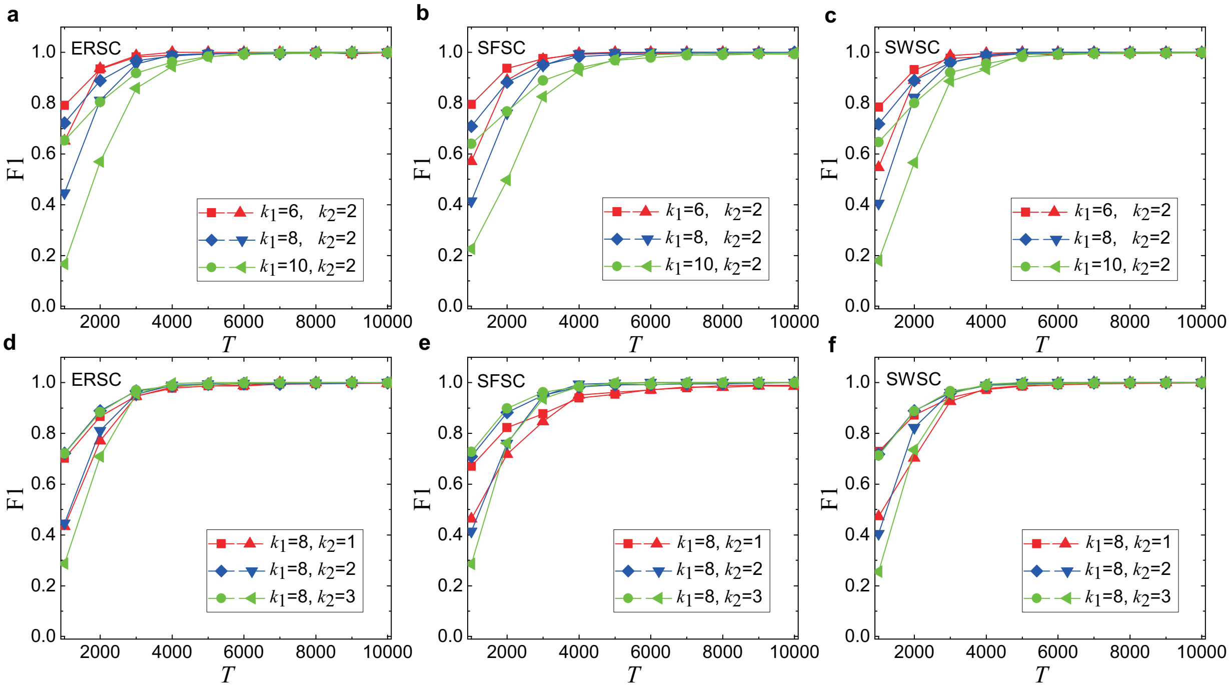

FIG. S5. Reconstruction performance for synthetic 2-simplicial complexes. Shown is F1 score as a function of the length T for three synthetic 2-simplicial complexes with size $N = 100$: ERSC - left column, SFSC - middle column, and SWSC - right column. In each panel, squares, diamonds and circles indicate the performance of reconstructing two-body connections while triangles with different orientations denote the performance of reconstructing three-body connections. Different values of the average degree are distinguished by colors. For $(k_1 = 6, k_2 = 2)$, $(k_1 = 8, k_2 = 2)$, $(k_1 = 10, k_2 = 2)$, $(k_1 = 8, k_2 = 1)$, and $(k_1 = 8, k_2 = 3)$, the parameter values are $(J_1, J_2) = (0.7, 1.2)$, $(0.8, 1.2)$, $(0.86, 1.2)$, $(0.8, 2.4)$ and $(0.8, 0.8)$, respectively. Other parameter values are $\rho_0 = 0.5$ and $\delta = 1/k_1$. Each data point is the result of averaging over five realizations.

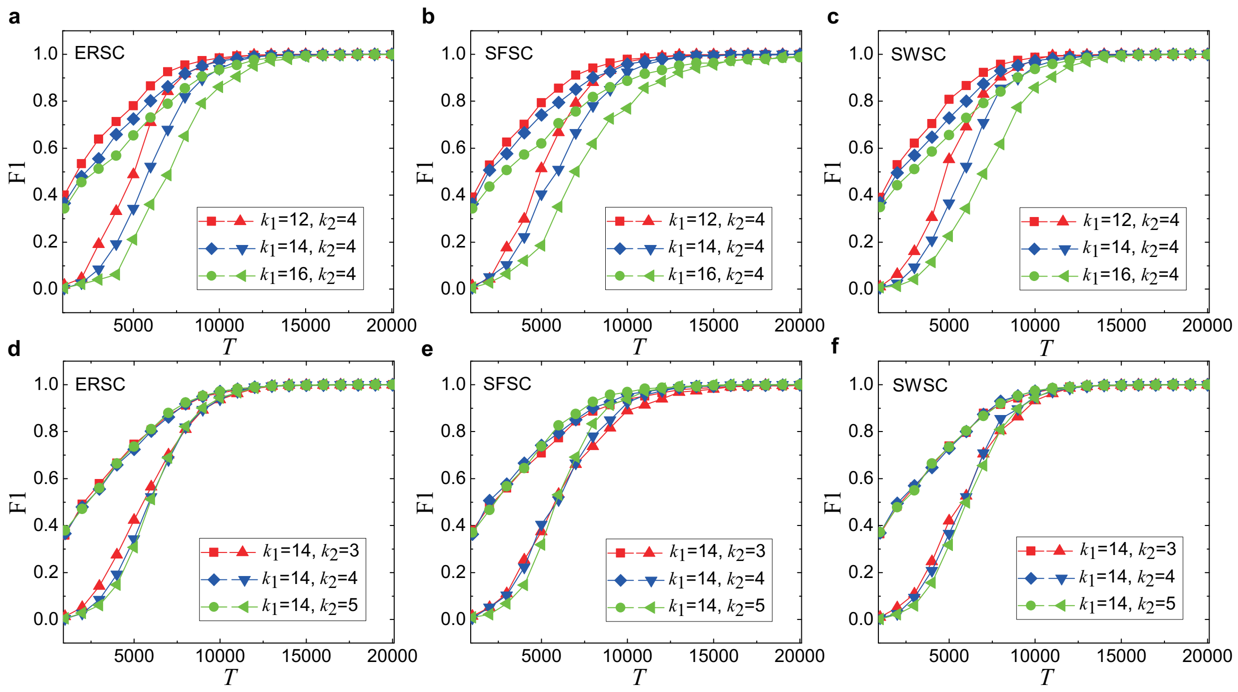

FIG. S6. Reconstruction performance for synthetic 2-simplicial complexes. The legends are the same as those in Fig. S5 except that the network size is $N = 200$. For $(k_1 = 12, k_2 = 4)$, $(k_1 = 14, k_2 = 4)$, $(k_1 = 16, k_2 = 4)$, $(k_1 = 14, k_2 = 3)$, and $(k_1 = 14, k_2 = 5)$, the parameter values are $(J_1, J_2) = (0.7, 1.2)$, $(0.76, 1.2)$, $(0.8, 1.2)$, $(0.76, 1.6)$ and $(0.76, 0.96)$, respectively. Other parameter values are $\rho_0 = 0.5$ and $\delta = 1/k_1$.

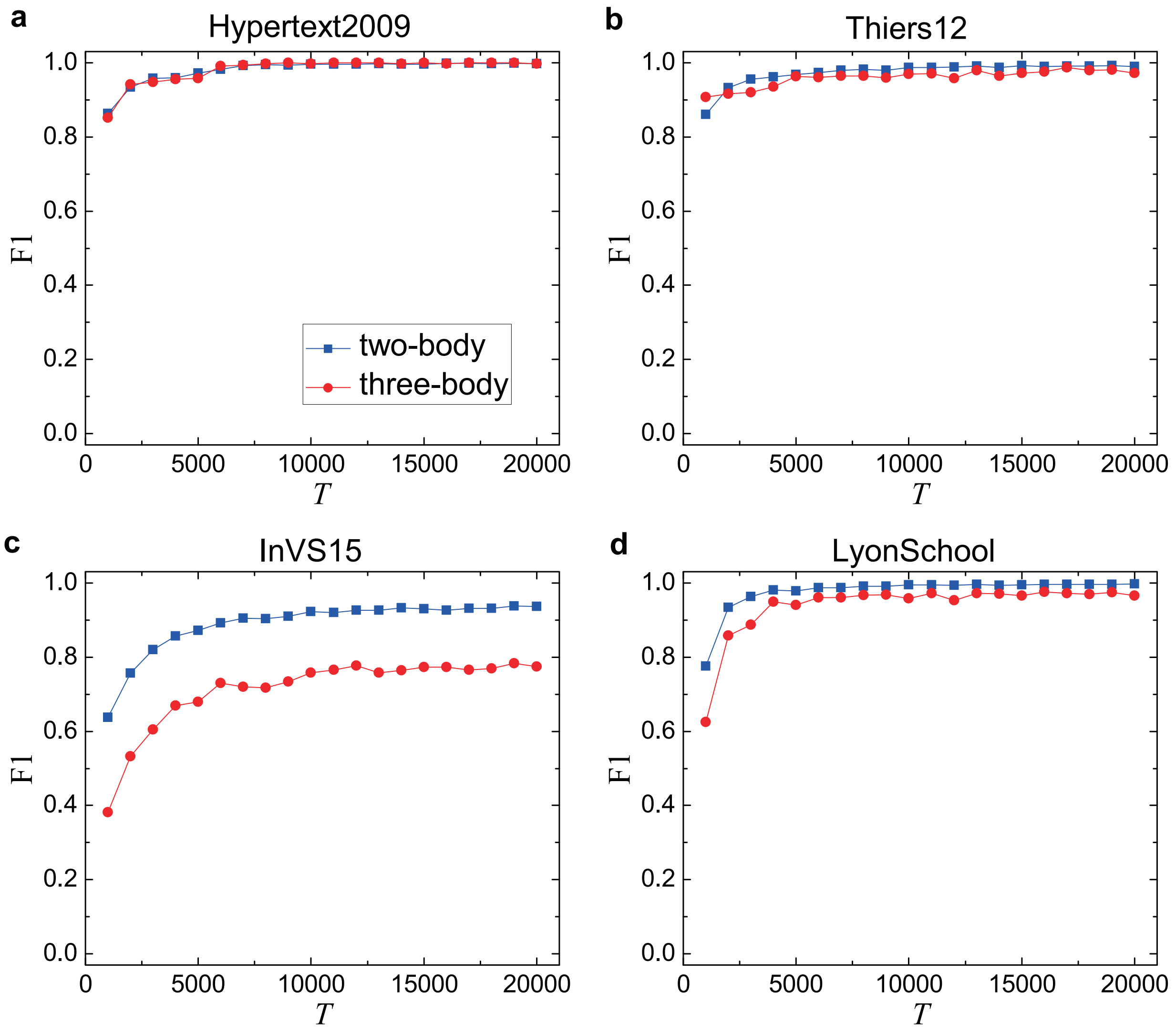

FIG. S7. Reconstruction performance for real-world 2-simplicial complexes. Shown is F1 score versus the time-series length T. The blue squares and red circles indicate the reconstruction performance of two-body and three-body connections, respectively. The parameter values are (**a**) $J_1 = 0.78$, $J_2 = 1.2$, (**b**) $J_1 = 0.8$, $J_2 = 1.2$, (**c**) $J_1 = 0.78$, $J_2 = 1.1$, (**d**) $J_1 = 0.66$, $J_2 = 1.1$. Other parameter values are $\rho_0 = 0.5$ and $\delta = 1/k_1$. Each data point is the result of averaging over five realizations.

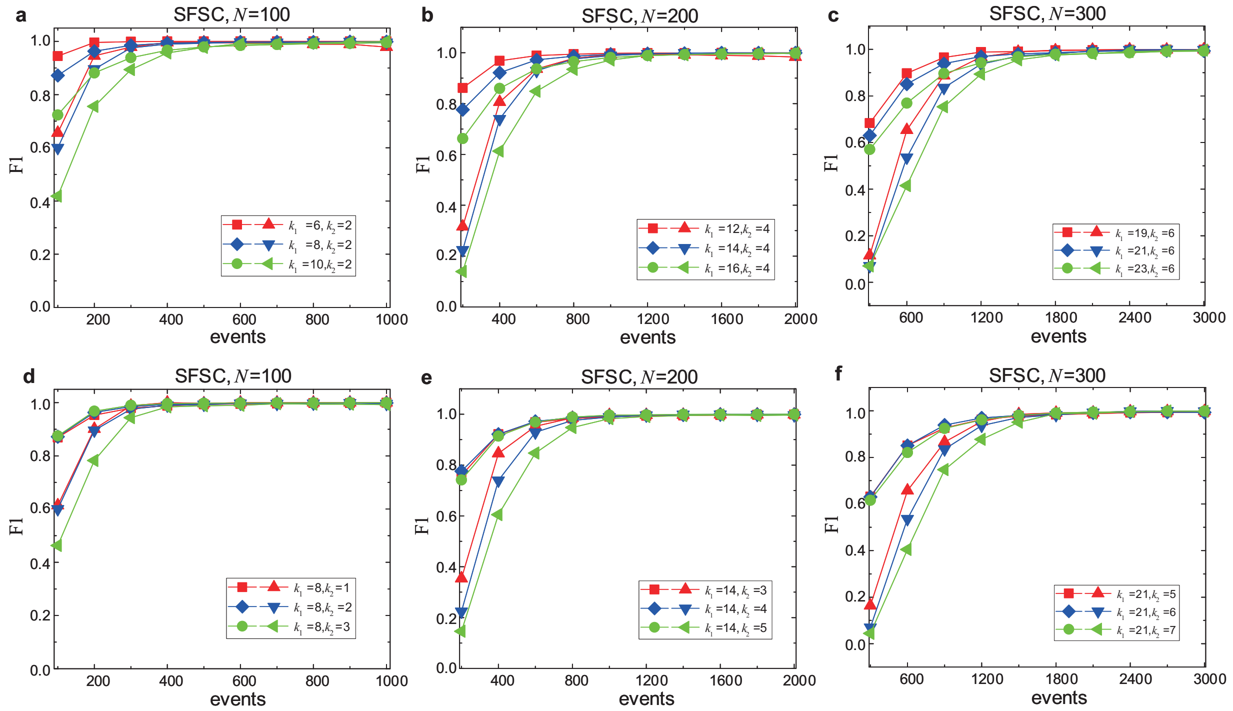

FIG. S8. F1 score as a function of the number of events for SFSC for (**a,d**) N = 100, α = 0.6, ω = 2, (**b,e**) N = 200, α = 0.7, ω = 2.2, and (**c,f**) N = 300, α = 0.75, ω = 2.3. In each panel, squares, diamonds and circles demonstrate the performance of reconstructing two-body connections, and triangles with different orientations depict the performance of reconstructing three-body connections. The average degrees are distinguished by colors. Other parameter values are $\rho_0 = 0.2$ and $\mu = 1$. Each data point is the result of averaging over five realizations.

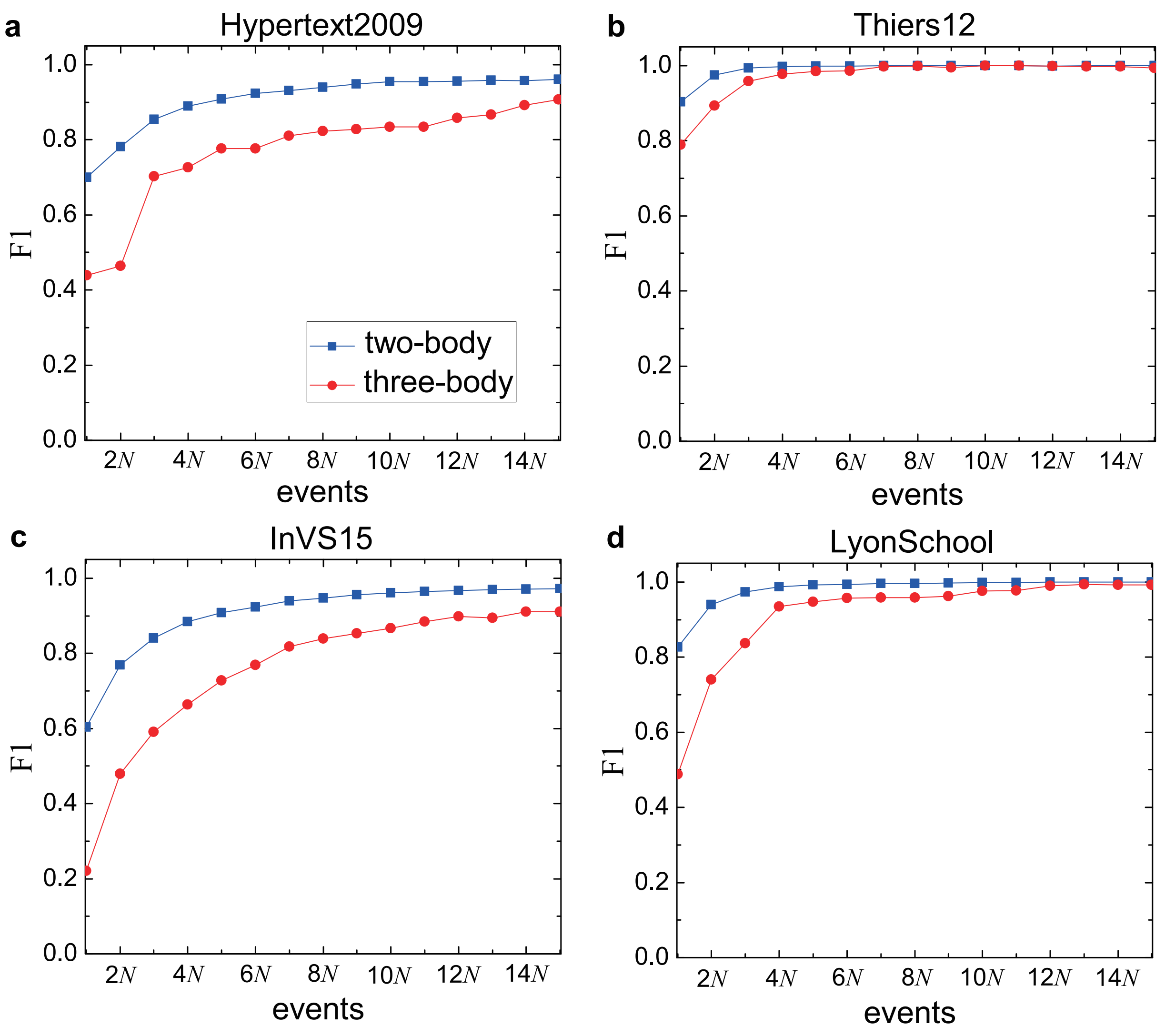

FIG. S9. F1 score as a function of the number of events for four real-world 2-simplicial complexes: (**a**) Hypertext2009, (**b**) Thiers12, (**c**)InVS15, (**d**) LyonSchool. In each panel, the blue squares and red circles demonstrate the performance of reconstructing two-body and three-body connections, respectively. Parameter values are $\alpha = 0.3$, $\omega = 1$, $\rho_0 = 0.2$ and $\mu = 1$. The "N" appeared in the abscissa denotes the size of the specific network. Each data point is the result of averaging over five realizations.

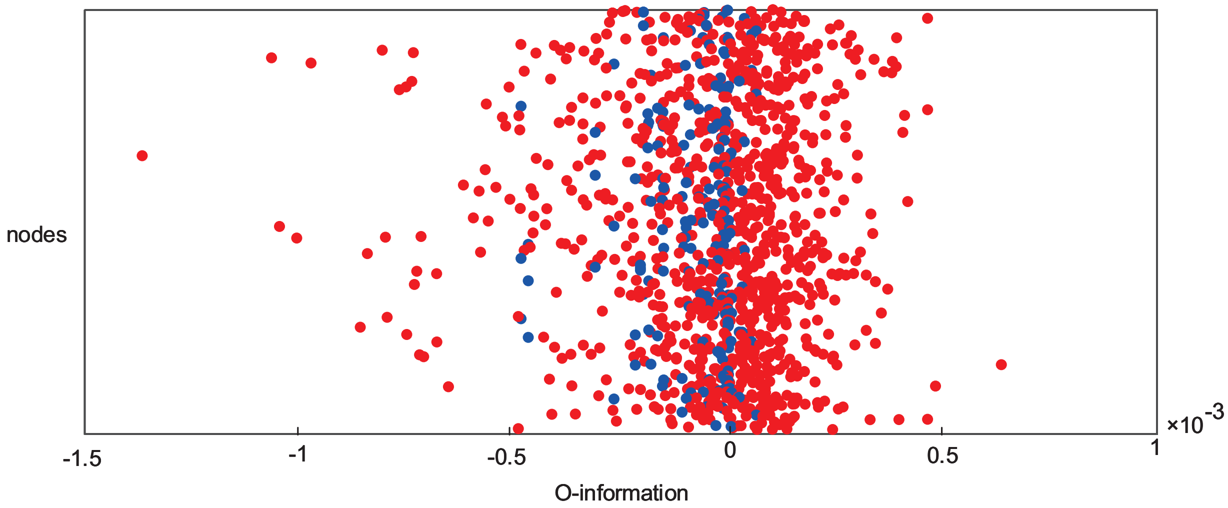

FIG. S10. Performance of O-information based method. Shown are the O-information values of any three points $\Omega(X^3)$ on ERSC. Each row corresponds to the O-information of one node. The blue and red dots denote the existent and nonexistent three-body connections, respectively. The parameter values are $N = 100$, $T = 10000$, $k_1 = 7$, $k_2 = 2$, $\alpha = 0.6$, $\omega = 2.1$, $\rho_0 = 0.2$ and $\mu = 1$. For each node, the number of all possible three-body interactions is about N^2. Because the number of existent three-body connections is very low, only 0.2% of the red dots are shown in each row for better visualization.

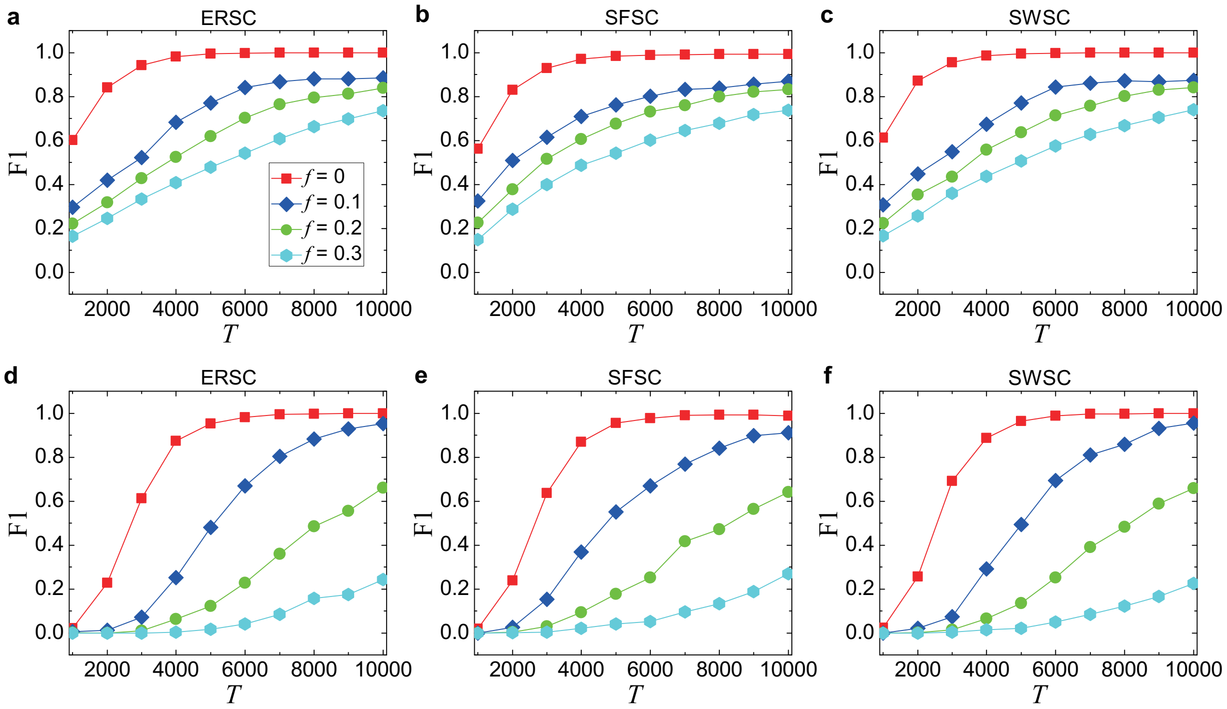

FIG. S11. Reconstruction performance under noise for synthetic 2-simplicial complexes. Shown is F1 score for different values of the flip ratio f as a function of the time-series length T for three synthetic 2-simplicial complexes: random simplicial complex (ERSC - left column), scale-free simplicial complex (SFSC - middle column), and small-world simplicial complex (SWSC - right column): (**a-c**) reconstructing two-body connections and (**d-f**) reconstructing three-body interactions. The results for different values of f are distinguished by symbols and colors. All simplicial complexes have the same size $N = 200$. Other parameter values are $k_1 = 12$, $k_2 = 4$, $\alpha = 0.8$, $\omega = 2.4$, $\rho_0 = 0.2$, and $\mu = 1$. Each data point is the result of averaging over five realizations.

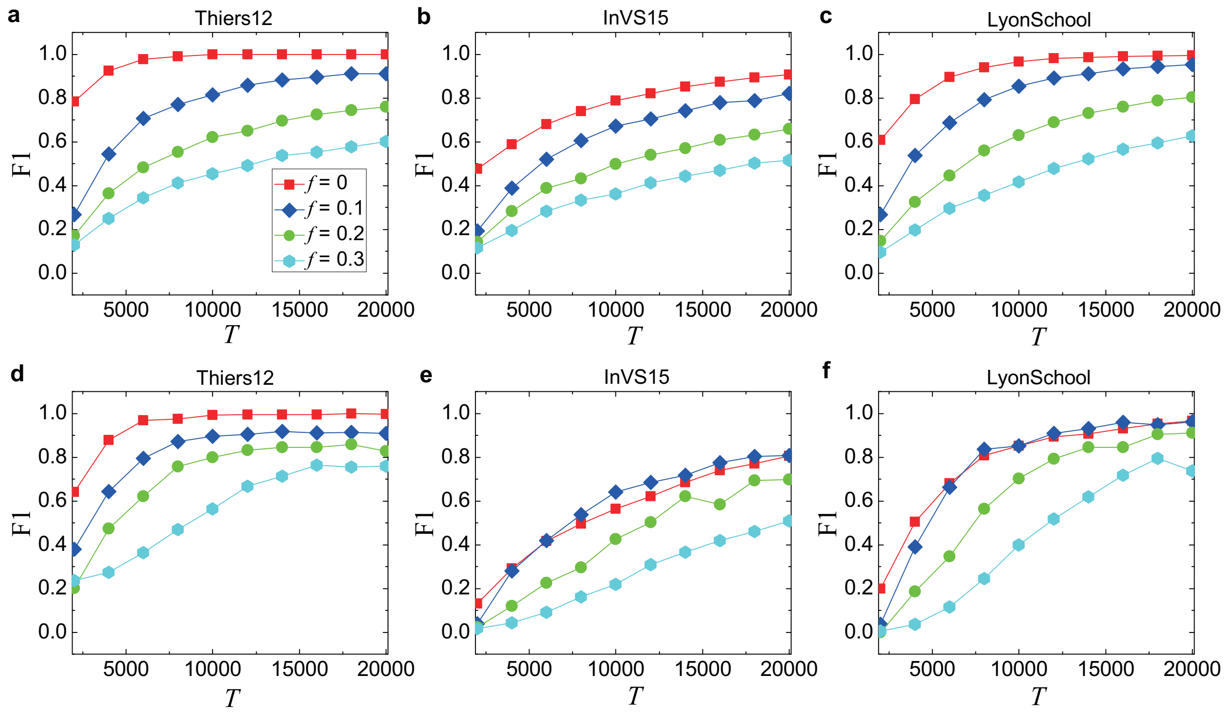

FIG. S12. Reconstruction performance under noise for real-world 2-simplicial complexes. Shown is F1 score for different values of the flip ratio f as a function of the time-series length T for (**a,d**) Thiers12, (**b,e**) InVS15, and (**c,f**) LyonSchool: (**a-c**) reconstructing two-body connections and (**d-f**) reconstructing three-body interactions. Parameter values are $\alpha = 0.3$, $\omega = 1$, $\rho_0 = 0.2$, and $\mu = 1$. Each data point is the result of averaging over five realizations.

VIII. SUPPLEMENTARY REFERENCES

[1] Dempster, A. P., Laird, N. M. & Rubin, D. B. Maximum likelihood from incomplete data via the EM algorithm. *J. R. Stat. Soc. Ser. B Method.* **39**, 1–22 (1977).

[2] Rosas, F. E., Mediano, P. A. M., Gastpar, M. & Jensen, H. J. Quantifying high-order interdependencies via multivariate extensions of the mutual information. *Phys. Rev. E* **100**, 032305 (2019).
